# Supplementary material for: MetaRibo-Seq measures translation in microbiomes
Source: Nat Commun. 2020 Jun 29;11:3268. doi: 10.1038/s41467-020-17081-z (PMC7324362; doi:10.1038/s41467-020-17081-z)
Supplement: Supplementary file 10 — Supplementary Data 7 [file 41467_2020_17081_MOESM10_ESM.zip › File2/Confidence_High_Taxonomy/220663_out.krona.html]

Javascript must be enabled to view this page.

members
magnitude
magnitudeUnassigned
count
unassigned
taxon
rank

220663\_out

104

superkingdom
104
2

phylum
976
104

class
200643
104

order
171549
104

102
1853231
family

5
genus

SRS024492\_contig\_number\_634SRS892882\_contig\_number\_5930SRS893366\_contig\_number\_1838SRS971275\_contig\_number\_14202SRS971276\_contig\_number\_10954
102
283168


SRS149181\_contig\_number\_contig-100\_97.168566
species
1897010
1

28118
92
species

SRS011084\_contig\_number\_30380SRS011134\_contig\_number\_19204SRS011239\_contig\_number\_2894SRS012849\_contig\_number\_1741SRS012969\_contig\_number\_16036SRS013098\_contig\_number\_10090SRS013521\_contig\_number\_7308SRS014979\_contig\_number\_9076SRS015431\_contig\_number\_39980SRS015663\_contig\_number\_29411SRS016095\_contig\_number\_1328SRS016335\_contig\_number\_35799SRS017191\_contig\_number\_12544SRS017247\_contig\_number\_8099SRS017433\_contig\_number\_15009SRS017521\_contig\_number\_1251SRS018133\_contig\_number\_14212SRS018623\_contig\_number\_14615SRS018836\_contig\_number\_16820SRS019068\_contig\_number\_55019SRS019161\_contig\_number\_27172SRS019397\_contig\_number\_11753SRS019601\_contig\_number\_30151SRS020328\_contig\_number\_12642SRS020394\_contig\_number\_contig-100\_564.85746SRS021219\_contig\_number\_contig-100\_1358.76938SRS021484\_contig\_number\_29998SRS022609\_contig\_number\_1575SRS023526\_contig\_number\_contig-100\_468.203395SRS024075\_contig\_number\_5867SRS024132\_contig\_number\_21851SRS024625\_contig\_number\_17409SRS043768\_contig\_number\_23530SRS044535\_contig\_number\_1981SRS045004\_contig\_number\_contig-100\_86.133480SRS045645\_contig\_number\_25446SRS045713\_contig\_number\_contig-100\_898.85442SRS046068\_contig\_number\_1600SRS046369\_contig\_number\_15809SRS047433\_contig\_number\_contig-100\_612.91519SRS048870\_contig\_number\_12243SRS049402\_contig\_number\_contig-100\_341.92363SRS049446\_contig\_number\_5741SRS049773\_contig\_number\_15650SRS049995\_contig\_number\_24571SRS050026\_contig\_number\_contig-100\_22.66680SRS050925\_contig\_number\_3574SRS050998\_contig\_number\_contig-100\_106.107SRS051031\_contig\_number\_376SRS051882\_contig\_number\_11762SRS052697\_contig\_number\_36524SRS053214\_contig\_number\_13403SRS053398\_contig\_number\_26048SRS054905\_contig\_number\_2693SRS058770\_contig\_number\_contig-100\_306.139705SRS063040\_contig\_number\_47679SRS063518\_contig\_number\_22905SRS063985\_contig\_number\_29113SRS076804\_contig\_number\_7531SRS077194\_contig\_number\_12529SRS077231\_contig\_number\_23389SRS077392\_contig\_number\_41751SRS077730\_contig\_number\_14886SRS077849\_contig\_number\_4671SRS078176\_contig\_number\_20478SRS078419\_contig\_number\_31446SRS078665\_contig\_number\_8600SRS097958\_contig\_number\_contig-100\_853.130170SRS100021\_contig\_number\_2910SRS103987\_contig\_number\_24599SRS1041037\_contig\_number\_11778SRS104311\_contig\_number\_30859SRS1054716\_contig\_number\_6233SRS1055022\_contig\_number\_15052SRS1055076\_contig\_number\_2304SRS140492\_contig\_number\_31989SRS140513\_contig\_number\_10970SRS142503\_contig\_number\_57822SRS142890\_contig\_number\_26359SRS143876\_contig\_number\_3283SRS144183\_contig\_number\_23521SRS144297\_contig\_number\_contig-100\_9927.87078SRS144362\_contig\_number\_contig-100\_1172.233441SRS144506\_contig\_number\_31156SRS147271\_contig\_number\_1841SRS148091\_contig\_number\_11425SRS148159\_contig\_number\_55964SRS148196\_contig\_number\_17433SRS148721\_contig\_number\_46466SRS149325\_contig\_number\_948SRS893279\_contig\_number\_contig-100\_487.43334SRS893383\_contig\_number\_23394

4
1637502
species

SRS020233\_contig\_number\_44878SRS075078\_contig\_number\_16455SRS1041118\_contig\_number\_8452SRS148424\_contig\_number\_contig-100\_1405.162950

2
815
family

genus
816
2

species

SRS146764\_contig\_number\_7388
820
1

1
28119
species

SRS019397\_contig\_number\_979
